# SNCA Overexpression Induces Apoptosis in Non–Small Cell Lung Cancer via Caspase‐Dependent Signaling Pathways

**DOI:** 10.1155/bmri/2222343

**Published:** 2026-04-15

**Authors:** Yong Qi Leong, Rhun Yian Koh, Christina Gertrude Yap, Soi Moi Chye, Khuen Yen Ng

**Affiliations:** ^1^ School of Pharmacy, Monash University Malaysia, Subang Jaya, Selangor, Malaysia, monash.edu.my; ^2^ Division of Biomedical Science and Biotechnology, School of Health Sciences, IMU University, Kuala Lumpur, Malaysia, imu.edu.my; ^3^ Jeffrey Cheah School of Medicine and Health Sciences, Monash University Malaysia, Subang Jaya, Selangor, Malaysia, monash.edu.my

**Keywords:** alpha-synuclein, apoptosis, lung cancer, overexpression, oxidative stress

## Abstract

The alpha‐synuclein (SNCA) gene is a Parkinson′s disease (PD)–associated gene that is found to be downregulated in non–small cell lung cancer (NSCLC). Aberrant SNCA expression exerts neurotoxicity in PD by disrupting mitochondrial function, promoting protein aggregation or oxidative stress, ultimately leading to neuronal cell death. Numerous studies have hypothesized that SNCA is a tumor suppressor gene, but the underlying mechanism remains elusive. In this study, the SNCA gene is delivered to NSCLC cells via transfection of a plasmid vector with carbonate apatite (CA) nanoparticles as the delivery vehicle. Biochemical assays including cytotoxicity assay, oxidative stress assay, flow cytometry, and caspase activity assay were performed to assess the effects of SNCA overexpression in NSCLC cells. SNCA‐overexpressed NSCLC cells were established using the optimized CA/plasmid complexes. SNCA overexpression promoted oxidative stress–induced cell death and apoptosis in both lung adenocarcinoma (LUAD) and lung squamous cell carcinoma (LUSC) cells. Additionally, SNCA overexpression activated distinct caspases′ activities in LUAD and LUSC. Besides, this study reveals that LUSC was more susceptible to the anticancer effects of SNCA overexpression than LUAD. As a result, SNCA overexpression promoted apoptosis under oxidative stress in NSCLC cells via distinct caspases′ activations.

## 1. Introduction

Non–small cell lung cancer (NSCLC) constitutes about 85% of total lung cancer occurrences, with lung adenocarcinoma (LUAD) being the predominant subtype at 40%, followed by lung squamous cell carcinoma (LUSC) at 25% and large cell carcinoma at 10% [1, 2]. Despite the standard of care and emerging targeted therapies, the overall survival rate in NSCLC patients remains low, as NSCLC is usually diagnosed at a late stage with a poor prognosis [3]. Thus, there is a need for the discovery of new therapeutic methods and effective molecular targets for NSCLC [3–5].

Remarkably, numerous epidemiological studies have demonstrated a significantly reduced risk of lung cancer among individuals with Parkinson′s disease (PD) [6–8]. Our previous meta‐analysis has revealed a significant negative association between lung cancer and PD patients (RR = 0.56, 95*%*CI = 0.48–0.66, *I* ^2^ = 91.3*%*, *p* < 0.001) [9]. Despite both diseases being known to be multifactorial involving influence from the genes and the environment, several studies have suggested that they share some common genetic features [10–12]. Several studies reported the aberrant expressions of PD‐associated genes, including alpha‐synuclein (SNCA), PTEN‐induced Kinase 1 (PINK1), parkin, parkinsonism‐associated deglycase (DJ‐1), leucine‐rich repeat Kinase 2 (LRRK2), F‐box Protein 7 (FBXO7), and ubiquitin C‐terminal hydrolase L1 (UCHL1) in lung cancer patients [11–13].

In PD, SNCA aggregates to form Lewy bodies, which exerts neurotoxicity and ultimately leads to neuronal cell death [14, 15]. SNCA facilitates the regulation of synaptic vesicles for neurotransmitter release, the control of endoplasmic reticulum (ER)–Golgi transport, mitochondrial balance, and molecular chaperone activity [16–18]. Other than that, a number of studies have indicated the correlation of SNCA to various cancers, including colorectal cancer [19], ovarian cancer [20], breast cancer [21], and melanoma [22]. Additionally, two studies have reported that SNCA is downregulated in LUAD via bioinformatic analyses [23, 24]. It is believed that SNCA is a tumor suppressor gene that its overexpression might inhibit NSCLC development. However, the anticancer role of SNCA as well as the underlying mechanisms and signaling pathways remains unknown.

Thus, this study is aimed at developing SNCA‐overexpressed NSCLC cell lines using carbonate apatite nanoparticles as a delivery vehicle. We also elucidated the underlying mechanisms by which SNCA overexpression elicits its anticancer effects in NSCLC cells.

## 2. Material and Methods

### 2.1. Cell Culture

LUAD cell line (A549), LUSC cell line (NCI‐H2170), and human control lung cell (MRC‐5) were obtained from the American Tissue Cell Culture Collection (ATCC; Manassas, Virginia, United States). The cells were cultured at 37°C with 5% CO_2_ in Dulbecco′s Modified Eagle Medium (DMEM, Gibco, Gaithersburg, Maryland, United States) supplemented with 10% fetal bovine serum (FBS, Gibco, Gaithersburg, Maryland, United States) and 1% penicillin/streptomycin (Gibco, Gaithersburg, Maryland, United States).

### 2.2. Plasmid Multiplication and Purification

Predesigned plasmid DNA specific for SNCA (*Homo sapiens* SNCA, transcript Variant 3, mRNA, EX‐G0543‐M29) and the corresponding negative control vector (EX‐NEG‐M29) were purchased from GeneCopoeia (Rockville, Maryland, United States). One microliter of SNCA plasmid was added into a microcentrifuge tube containing 50 *μ*L ECOS 101 (DH5*α*) competent cells (Yeastern Biotech Co. Ltd., Taiwan). The tube was incubated at 42°C for 1 min. After incubation, the tube was buried in ice immediately. Followingly, the mixture was added onto the LB agar (Solarbio Life Science, China) containing 1 mg/mL ampicillin (Sigma‐Aldrich, United States) for streaking. The plate was incubated overnight at 37°C. The next day, several colonies were picked and added to the LB broth containing 1 mg/mL ampicillin, allowing the bacteria to grow. The steps were repeated for the negative control vector. Then, the bacterial suspensions were harvested, and the plasmids were purified by using the FavorPrep Endotoxin‐Free Plasmid DNA Extraction Midi Kit (Favorgen, Taiwan). The plasmids were then quantified by using the NanoQuant microplate reader (SpectraMax M Series, United States).

### 2.3. Formulation of CA/Plasmid Complexes

CA nanoparticles were fabricated according to the protocol established by Chowdhury et al. [25, 26]. Briefly, 100 mL of the buffer solution was prepared by using 1.35 g DMEM powder and 0.37 g sodium bicarbonate (Sigma‐Aldrich, United States). Then, the pH was adjusted to 7.4 by using 1 M hydrochloric acid (Sigma‐Aldrich, United States). Followingly, the bicarbonate‐buffered DMEM solution was filtered by a 0.22 *μ*m syringe filter. Eight microgram plasmid DNA and 5 mM exogenous calcium chloride dihydrate (CaCl_2_•2H_2_O, Sigma‐Aldrich, United States) were then added into a 1.5‐mL tube containing 1 mL of bicarbonate‐buffered DMEM. Then, the CA/plasmid complexes were formulated after the incubation for 30 min at 37°C, followed by the addition of 10% FBS to prevent further aggregation.

### 2.4. Validation of SNCA‐Overexpressed Cell Models by Using Fluorescent Microscopy and Western Blot Analysis

MRC‐5, A549, and NCI‐H2170 were seeded onto 6 cm culture dishes until the cells′ confluency reached around 70%. CA/plasmid complexes were synthesized as described in Section 2.3. The cells were treated with CA nanoparticles only (control), CA/pEX‐NEG‐M29 complexes (empty vector), and CA/pEX‐G0543‐M29 (SNCA), respectively. After 48‐h incubation, the cells were washed with 5 mM ethylenediaminetetraacetic acid (EDTA) disodium salt (Sigma‐Aldrich, United States) in phosphate‐buffered saline (PBS, Bio Basic Inc., Canada) to remove the extracellular particles. The enhanced green fluorescent protein (eGFP) expressions were detected at 100× magnification using an Eclipse Ti‐U inverted microscope to study the effect of transfection efficiency. The acquisition and processing of both bright field and fluorescence images were performed using NIS‐Elements software. Afterwards, cell lysates were extracted with ice‐cold RIPA buffer (#ab156034, Abcam, United States) containing 1 mM phenylmethanesulfonyl fluoride (Sigma‐Aldrich, United States) and protease inhibitor (1:200; Calbiochem, Merck, Germany) and phosphatase inhibitor (1:100; Calbiochem, Merck, Germany). The protein concentrations of cell lysates were measured using the Quick Start Bradford protein assay kit (Bio‐Rad Laboratories, United States). The color intensity was measured at 595 nm using a UV/Vis spectrophotometer. Forty micrograms of protein samples and 6 *μ*L of ExcelBand enhanced three‐color high‐range protein marker (SMOBIO Technology Inc., Taiwan) were then resolved by SDS–polyacrylamide gel electrophoresis (SDS‐PAGE) and transferred onto polyvinylidene fluoride (PVDF) membranes (Bio‐Rad Laboratories, United States). The membrane was blocked with 5% bovine serum albumin (BSA, Nacalai Tesque Inc., Japan). Then, the membrane was incubated with SNCA (sc‐7011‐R; Santa Cruz Biotechnology Inc., United States) and beta‐actin (sc‐47778; Santa Cruz Biotechnology Inc., United States) primary antibodies, overnight at 4°C. All primary antibodies were prepared in a 1:1000 dilution using blocking buffer and sodium azide (Sigma‐Aldrich, United States). Following three consecutive 5‐min washes in TBS‐T, horseradish peroxidase (HRP)–conjugated antirabbit and antimouse (Cell Signaling Technology Inc., United States) secondary antibodies (1:10,000) were added. Following three more 5‐min washes, the blots were developed using a chemiluminescence kit, SuperSignal West Femto Maximum Sensitivity Substrate (Thermo Fisher Scientific, United States). Finally, the band densities were detected using Amersham Imager 600 (GE Healthcare Life Sciences, United Kingdom).

### 2.5. Measurement of Cell Cytotoxicity

The cytotoxic effects of SNCA overexpression against NSCLC cell lines were tested by the 3‐(4,5‐dimethylthiazol‐2‐yl)‐2,5‐diphenyltetrazolium bromide (MTT) colorimetric assay. In detail, MRC‐5, A549, and NCI‐H2170 were seeded onto 96‐well plates until the cells′ confluency reached around 70%. Then, the cells were treated with formulated CA/plasmid complexes as described in Section 2.3. After 48 h of transfection, 20 *μ*L of 5 mg/mL MTT solution (Calbiochem, United States) was added to each well. After that, the 96‐well plates were incubated for another 4 h at 37°C. The media containing MTT solution was then replaced by 100 *μ*L of dimethyl sulfoxide (DMSO; Sigma‐Aldrich, United States) to dissolve the purple formazan crystals. A microplate reader (SpectraMax M Series, United States) was used to measure the absorbance values at a wavelength of 570/630 nm. The cell viability was calculated using the following formula:

Cell viability %=absorbance of treated cellsabsorbance of untreated cells×100%

### 2.6. Detection of Intracellular Reactive Oxygen Species (ROS)

To detect ROS generation in the NSCLC cells, 2 ^′^,7 ^′^‐dichlorofluorescein diacetate (DCFH‐DA; Sigma‐Aldrich, United States) was used. MRC‐5 and NCI‐H2170 were seeded on 24‐well plates at a density of 4.5 × 10^4^ cells/mL, whereas A549 was seeded at 3.5 × 10^4^ cells/mL. After overnight culturing, the cells were transfected with CA/plasmid complexes for 48 h at 37°C and 5% CO_2_. Afterwards, the cells were trypsinized and centrifuged at 1500 rpm for 5 min. The supernatant was discarded and replaced with 500 *μ*L PBS. One hundred microliters of the resuspended samples was added onto 96‐well black plates. Then, each well was treated with 100 *μ*L PBS containing DCFH‐DA. The plate was read at 0, 10, 20, and 30 min at 485 nm with reference to 535 nm using a plate reader where the plate was incubated at 37°C during the intervals. The results were then normalized to the number of cells counted using the trypan blue staining method using Microsoft Excel with the formulae below:

Normalization=average optical density of samplenumber of cells counted 

ROS fold change=ROS generated from sampleROS generated from control

### 2.7. Cell Cycle Analysis Through Flow Cytometry

After 48 h of transfection, the cells were trypsinized and centrifuged at 1500 rpm for 5 min. After centrifugation, the supernatant was discarded, and each cell pellet was fixed with 1 mL ice‐cold 70% ethanol and stored overnight at 4°C. The next day, each cell pellet was washed twice with 1 mL PBS at 3000 rpm for 5 min to remove excessive ethanol. Then, 500 *μ*L staining solution containing 20 *μ*g/mL PI (BD Biosciences, San Jose, California, United States) and 15 *μ*g/mL RNase (Merck, Germany) was added to each cell pellet, and the resultant mixture was incubated at room temperature for 30 min. All the samples were analyzed by passaging through FACSCalibur employing the BD CellQuest Pro software.

### 2.8. Annexin V–FITC Analysis Through Flow Cytometry

After 48 h of transfection, the cells were harvested and resuspended in 400 *μ*L of binding buffer. The cells were then stained with 5 *μ*L of Annexin V–FITC (BD Biosciences, United States) and PI (BD Biosciences), respectively, and mixed evenly. Subsequently, the samples were left to stand in the dark at room temperature for 15 min and then analyzed for apoptosis by flow cytometry through FACSCalibur employing the BD CellQuest Pro software within 1 h.

### 2.9. Measurement of Caspase Activities

To study the cell death mechanisms triggered by SNCA transfection in the lung cancer cells, the Caspase‐Glo 3/7, 8, and 9 assay kits (Promega Corporation, United States) were used according to the protocol provided by the manufacturer. Briefly, MRC‐5, A549, and NCI‐H2170 cells were plated in 96‐well white plates and 96‐well transparent plates, respectively. After 48 h of plasmid transfection, 50 *μ*L of the caspase‐3/7, caspase‐8, or caspase‐9 reagents was added to the wells containing cells. Subsequently, the mixture was incubated at room temperature for 30 min on a shaker. Finally, the luminescence of each sample was quantified in a plate‐reading luminometer. On top of that, expression levels of caspase‐2, caspase‐6, and caspase‐12 were also studied using ELISA kits (FineTest Biotech Inc., Wuhan, China). Cell lysates were harvested using lysis buffer and quantified as described in Section 2.8. According to the manufacturer′s protocol, 100 *μ*L of diluted samples of 30 *μ*g proteins was added onto 96‐well plates, followed by biotin‐labeled antibody, HRP‐streptavidin conjugate, TMB substrate, and stop solution, respectively. Finally, the absorbance was measured immediately at 450 nm in a microplate reader, and the concentration of each sample was calculated.

### 2.10. Statistical Analysis

All experiments were performed in triplicate unless otherwise stated. Results were displayed as mean ± standard error of mean (SEM) from at least three independent experiments. All statistical analyses were assessed with two‐way ANOVA using GraphPad Prism software (Version 9.5.0), followed by Tukey′s post hoc test. *p* values < 0.05 were considered statistically significant.

## 3. Results

### 3.1. SNCA Was Upregulated in MRC‐5, A549, and NCI‐H2170 Cells

The presence of green fluorescent signals in all three MRC‐5, A549, and NCI‐H2170 demonstrated the successful transfection of plasmids using CA nanoparticles as the delivery vehicle (Supporting Information 1: Figure S1). Western blot showed that SNCA protein levels increased significantly by 3.8‐, 2.6‐, and 3.6‐fold in SNCA‐transfected MRC‐5, A549, and NCI‐H2170, respectively, as compared to empty vector–transfected cells (*p* < 0.05, Figure 1).

Figure 1Validations of transfection in MRC‐5, A549, and NCI‐H2170 cells. (a) Protein expression levels of SNCA in MRC‐5, A549, and NCI‐H2170 were confirmed by western blot analysis against beta‐actin, the housekeeping gene. Molecular weights of SNCA and beta‐actin are 43.5 and 42 kDa, respectively. Quantified results of western blotting of SNCA‐overexpressed (b) MRC‐5, (c) A549, and (d) NCI‐H2170. Lanes 1, 4, and 7: control; Lanes 2, 5, and 8: empty vector–transfected cells; Lanes 3, 6, and 9: SNCA‐overexpressed cells. Blots are representative of four independent experiments. Negligible expression in CA nanoparticles only and empty vector–transfected cells compared to SNCA‐transfected cells confirmed that the protein expression of SNCA has been overexpressed in the three cell lines. Data were expressed as mean ± SEM of four independent experiments (*n* = 4). ∗∗ represents statistical significance (two‐way ANOVA followed by Tukey′s post hoc test,  ^∗∗^ *p* < 0.01,  ^∗∗∗∗^ *p* < 0.0001). CA: carbonate apatite; SEM: standard error of mean; SNCA: alpha‐synuclein.

**Figure:**
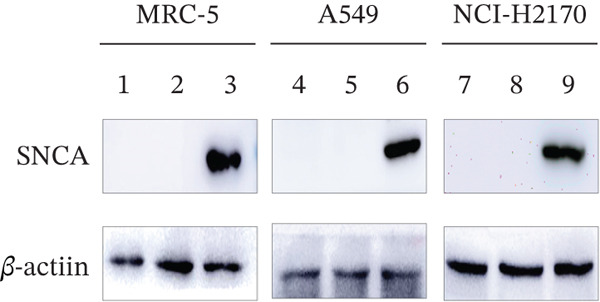
(a)

**Figure:**
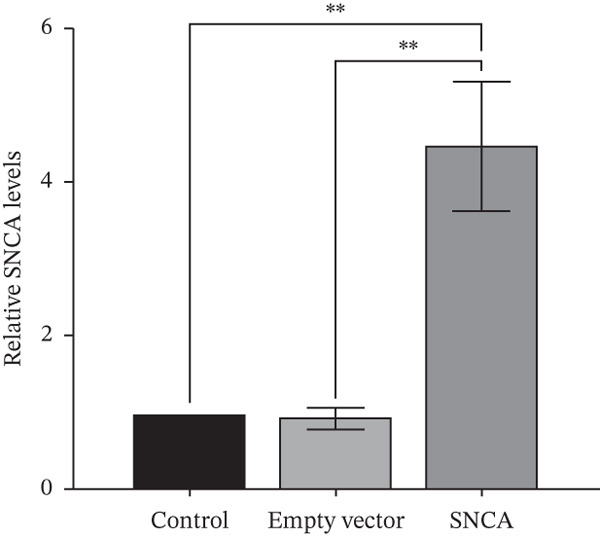
(b)

**Figure:**
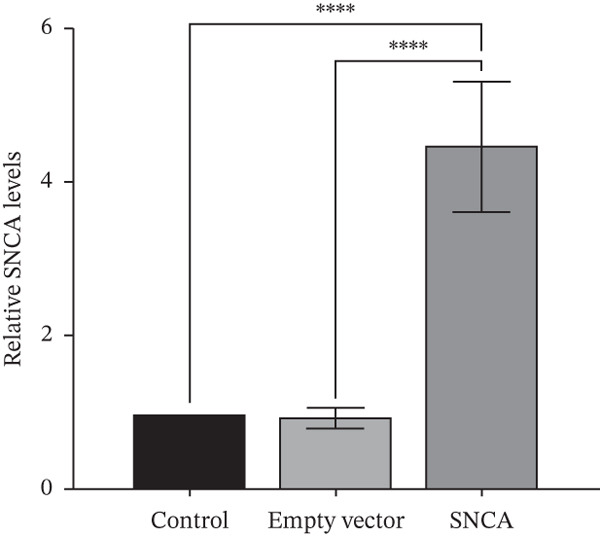
(c)

**Figure:**
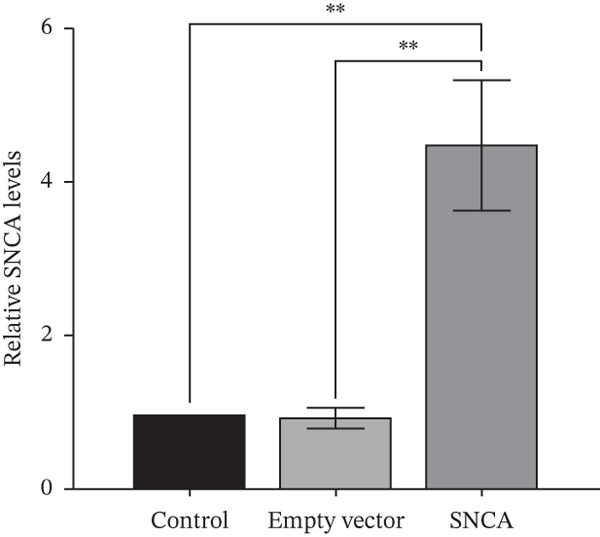
(d)

### 3.2. SNCA Overexpression Promoted Cell Cytotoxicity and Upregulation of ROS

In PD, it is believed that SNCA accumulation promotes its aggregation and exerts neurotoxicity [27]. Increased levels of ROS have been implicated in inflammation, cellular damage, and DNA mutation, which eventually promotes oxidative stress–induced cell death [28, 29]. To determine the biological significance of SNCA overexpression in NSCLC cells, MTT and DCFH‐DA assays were performed to examine the cell viability and ROS levels in SNCA‐overexpressed cells. As shown in Figure 2a, SNCA overexpression resulted in 32%, 8%, and 19% decline in cell viability in MRC‐5, A549, and NCI‐H2170, respectively, when compared to empty vector–transfected cells (*p* < 0.05 vs. empty vector–transfected cells). In this study, SNCA overexpression appeared to suppress cell growth and impose minimal but significant cytotoxic effects on NSCLC cells. Subsequently, the results also showed that intracellular ROS levels in the SNCA‐overexpressed MRC‐5, A549, and NCI‐H2170 cells were significantly increased by 52%, 62%, and 64%, respectively, when compared to empty vector–transfected cells (*p* < 0.05 vs. empty vector–transfected cells; Figure 2b). Together, the results suggested that SNCA overexpression has exerted oxidative stress on the NSCLC cells and is likely responsible for the observed cell death.

Figure 2Detection of cell viability and ROS levels of SNCA‐overexpressed MRC‐5, A549, and NCI‐H2170 using MTT assay and DCFH‐DA assay. (a) Cell viabilities of MRC‐5, A549, and NCI‐H2170 after 48‐h transfection with CA/SNCA complexes compared to a control group treated with CA nanoparticles only and cells transfected with CA/empty vector complexes. (b) Determination of induced reactive oxygen species of SNCA‐overexpressed MRC‐5, A549, and NCI‐H2170 cells. Data were expressed as mean ± SEM of five independent experiments performed in triplicate (*n* = 5). ∗ represents statistical significance (two‐way ANOVA followed by Tukey′s post hoc test,  ^∗^ *p* < 0.05,  ^∗∗^ *p* < 0.01,  ^∗∗∗^ *p* < 0.001,  ^∗∗∗∗^ *p* < 0.0001). DCFH‐DA: 2 ^′^,7 ^′^‐dichlorofluorescein diacetate; CA: carbonate apatite; SEM: standard error of mean; SNCA: alpha‐synuclein.

**Figure:**
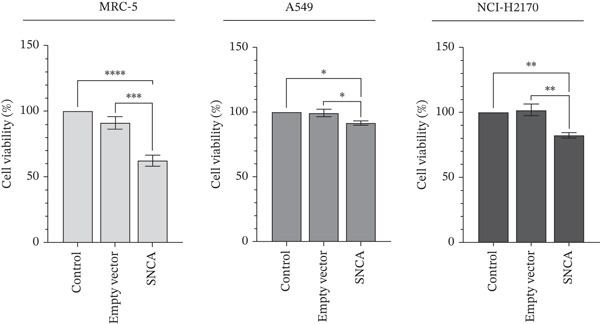
(a)

**Figure:**
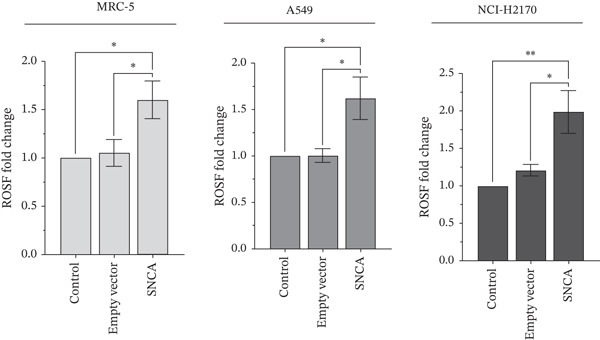
(b)

### 3.3. SNCA Overexpression Increased Sub‐G1 Cell Population

To study the antiproliferative mechanism of SNCA in MRC‐5 and NSCLC cells, we tested whether SNCA overexpression affected the cell cycle of the cells. The inhibitory effect of SNCA was assessed using cell cycle analysis. The results, as shown in Figure 3a and Supporting Information 2: Figure S2A, showed a significant increase in cell population in the sub‐G1 phase (7.8%, *p* < 0.05 vs. empty vector–transfected cells) and a significant decrease in cell population in the S phase (9.3%, *p* < 0.05 vs. empty vector–transfected cells) of the MRC‐5 cells. Similar to MRC‐5 cells, A549 and NCI‐H2170 cells also demonstrated changes in cell populations, albeit these changes were more substantial. Cell population in the sub‐G1 phase increased significantly in both SNCA‐overexpressed A549 and NCI‐H2170 cells as compared to their corresponding empty vector–transfected group by 56% and 185% (Figure 3b,c; Supporting Information 2: Figure S2B,C). Subsequently, cell populations were decreased significantly by 2.8% and 16.9% in G0/G1 and G2/M phases, respectively, in SNCA‐overexpressed A549 cells when compared to their corresponding empty vector–transfected group (Figure 3b, Supporting Information 2: Figure S2B). Besides, the cell populations were also decreased significantly by 10.2% (*p* < 0.05 vs. empty vector–transfected cells) and 25.2% (*p* < 0.05 vs. empty vector–transfected cells) in G0/G1 and G2/M phases, respectively, in SNCA‐overexpressed NCI‐H2170 cells (Figure 3c, Supporting Information 2: Figure S2C).

Figure 3Quantification analysis of cell cycle analysis is presented in a bar chart after 48 h of transfection with CA nanoparticles only, CA/empty vector, or CA/SNCA complexes in (a) MRC‐5, (b) A549, and (c) NCI‐H2170 cells. Data were expressed as mean ± SEM of three independent experiments performed in triplicate (*n* = 3). ∗ represents statistical significance (two‐way ANOVA followed by Tukey′s post hoc test,  ^∗^ *p* < 0.05,  ^∗∗^ *p* < 0.01,  ^∗∗∗^ *p* < 0.001,  ^∗∗∗∗^ *p* < 0.0001). CA: carbonate apatite; SEM: standard error of mean; SNCA: alpha‐synuclein.

**Figure:**
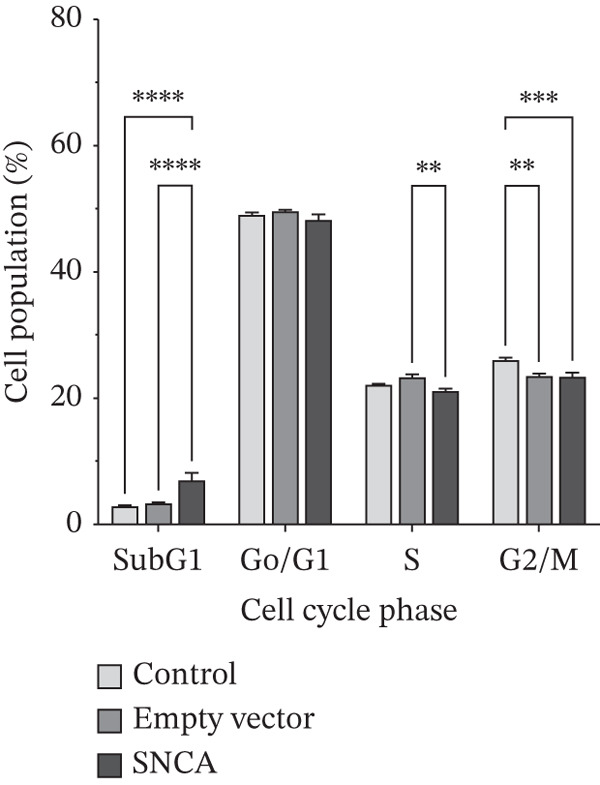
(a)

**Figure:**
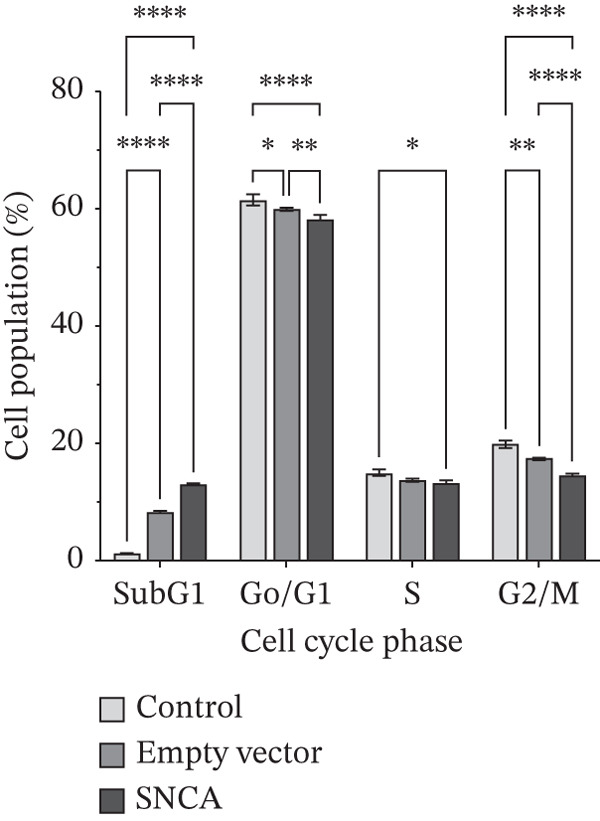
(b)

**Figure:**
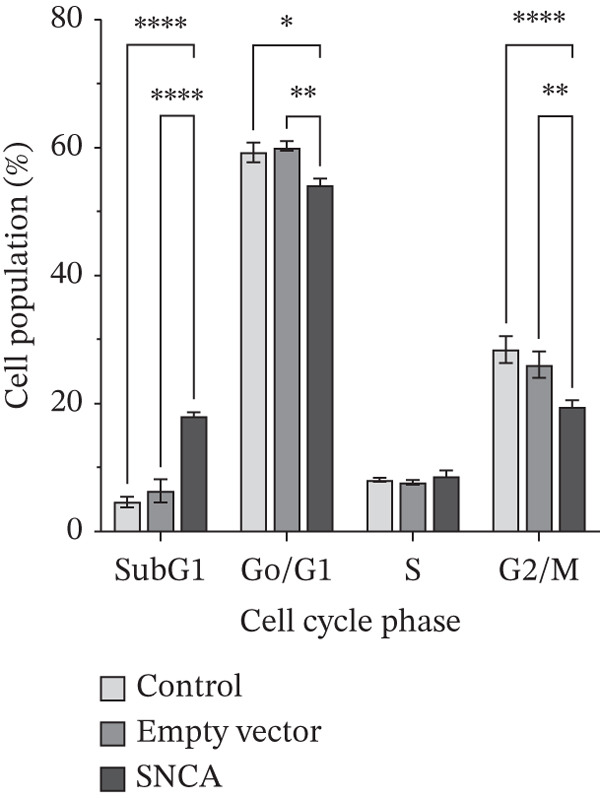
(c)

### 3.4. SNCA Overexpression Induced Apoptosis

In this experiment, the apoptosis induction effect of SNCA overexpression on MRC‐5, A549, and NCI‐H2170 cells was examined. Dot plot graphs depict different cell populations based on their characteristics, including viable cells in the lower left quadrant, early phase apoptotic cells in the lower right quadrant, late‐phase apoptotic or deceased cells in the upper right quadrant, and necrotic cells in the upper left quadrant (Supporting Information 3: Figure S3). As shown in Figure 4a,b and Supporting Information 3: Figure S3A,B, the early apoptotic cell population of SNCA‐overexpressed MRC‐5 and A549 in the lower right quadrant was increased to 37.82*%* ± 1.07*%* (*p* < 0.05 vs. control cells, *p* < 0.05 vs. empty vector–transfected cells) and 34.94*%* ± 6.28*%* (*p* < 0.05 vs. control cells), respectively. Likewise, the healthy cell population at the lower left quadrant decreased significantly by 23% in SNCA‐overexpressed MRC‐5 cells (*p* < 0.05 vs. empty vector–transfected cells), whereas the healthy cell population decreased by 13% in SNCA‐overexpressed A549 cells (*p* < 0.05 vs. empty vector–transfected cells) (Figure 4a,b; Supporting Information 3: Figure S3A,B). Negligible changes were found in the late apoptotic cell population in the upper right quadrant in SNCA‐overexpressed MRC‐5 and A549 cells when compared to the empty vector–transfected cells (Figure 4a,b; Supporting Information 3: Figure S3A,B). In addition, the percentage of late apoptotic cells for SNCA‐overexpressed NCI‐H2170 in the upper right quadrant increased significantly to 11.86*%* ± 0.19*%* (*p* < 0.05 vs. control cells, *p* < 0.05 vs. empty vector–transfected cells) with a 16% drop of the cell population (*p* < 0.05 vs. empty vector–transfected cells) at the lower right quadrant (early apoptotic cells) (Figure 4c and Supporting Information 3: Figure S3C).

Figure 4Quantification analysis of Annexin V–FITC analysis is presented in a bar chart after 48 h of transfection with CA nanoparticles only, CA/empty vector, or CA/SNCA complexes in (a) MRC‐5, (b) A549, and (c) NCI‐H2170 cells. Data were expressed as mean ± SEM of three independent experiments performed in triplicate (*n* = 3). ∗ represents statistical significance (two‐way ANOVA followed by Tukey′s post hoc test,  ^∗^ *p* < 0.05,  ^∗∗^ *p* < 0.01,  ^∗∗∗^ *p* < 0.001,  ^∗∗∗∗^ *p* < 0.0001). CA: carbonate apatite; SEM: standard error of mean; SNCA: alpha‐synuclein.

**Figure:**
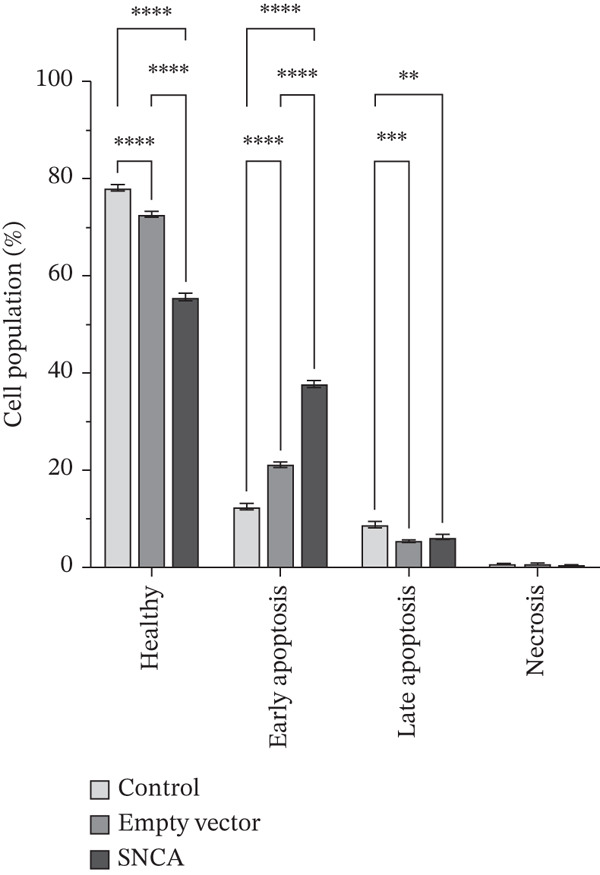
(a)

**Figure:**
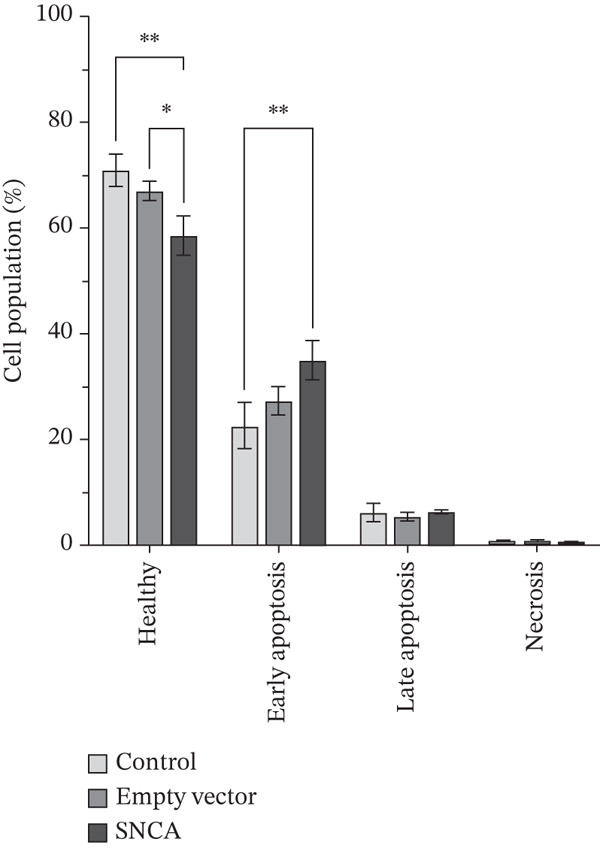
(b)

**Figure:**
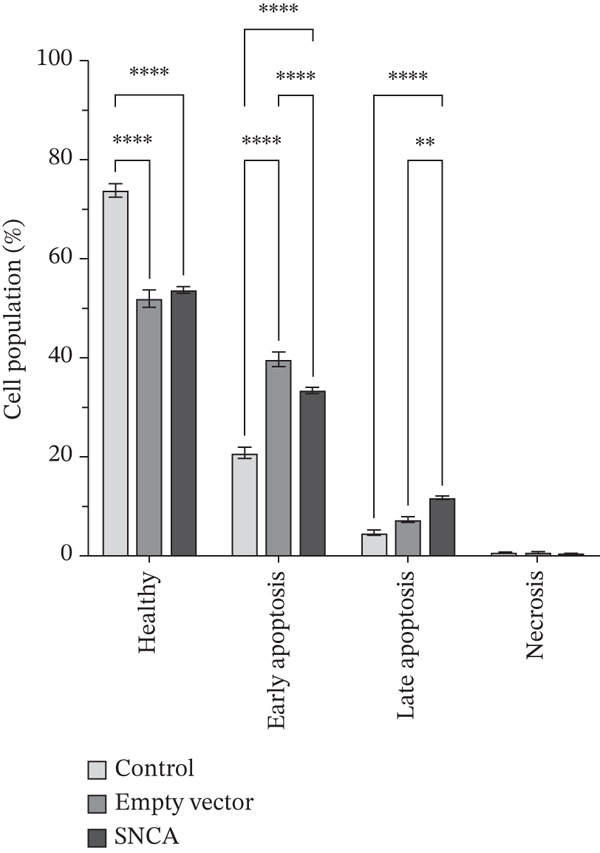
(c)

### 3.5. Caspase Activities in SNCA‐Overexpressed MRC‐5, A549, and NCI‐H2170 Cells

**Figure 5:**
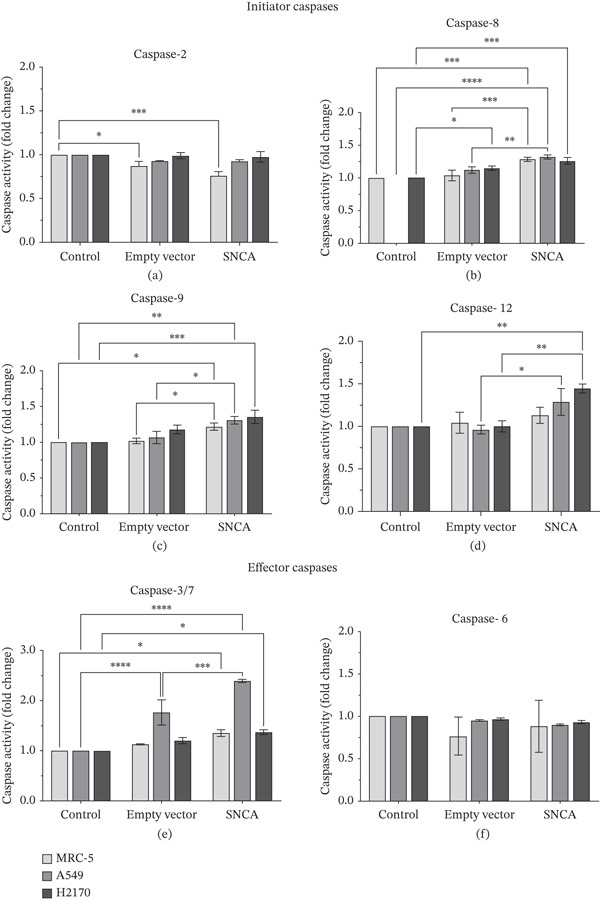
Expression levels of both initiator and effector caspases. (a) Caspase‐2, (b) caspase‐8, (c) caspase‐9, (d) caspase‐12, (e) caspase‐6, and (f) caspase‐3/7 activities in SNCA‐overexpressed MRC‐5, A549, and NCI‐H2170 were determined. Data were expressed as mean ± SEM of three independent experiments performed in triplicate (*n* = 3). ∗ represents statistical significance (two‐way ANOVA followed by Tukey′s post hoc test,  ^∗^ *p* < 0.05,  ^∗∗^ *p* < 0.01,  ^∗∗∗^ *p* < 0.001,  ^∗∗∗∗^ *p* < 0.0001). SEM: standard error of mean; SNCA: alpha‐synuclein.

Most cancer cells display increased levels of constitutive DNA damage due to replication stress and mitotic abnormalities that ultimately lead to cell death via apoptosis [30]. Caspase activity is the main factor in the regulation of the apoptosis mechanism. Caspases play different roles in apoptosis initiation. The effector caspase (caspase‐3/7 and caspase‐6) will be activated by initiator caspases (caspase‐2, caspase‐8, caspase‐9, and caspase‐12) [31, 32]. We therefore aimed to determine the role of SNCA overexpression in modulating caspase activities toward cancer cell behavior under ambient conditions. To assess the effect of SNCA overexpression on the activities of the main caspases (caspase‐3/7, caspase‐8, and caspase‐9) in MRC‐5 and NSCLC cells, the caspase activities were evaluated with a cell‐based homogeneous Caspase‐Glo assay kit. Moreover, the other caspase activities including caspase‐2, caspase‐6, and caspase‐12 were also studied using a caspase ELISA kit on SNCA‐overexpressed cells. There was no significant change in the activity level of caspase‐2 in SNCA‐overexpressed cells (Figure 5a). Furthermore, the results demonstrated that caspase‐8 activity increased significantly by 24% and 18% in SNCA‐overexpressed MRC‐5 and A549 cells, respectively (*p* < 0.05 vs. empty vector–transfected cells; Figure 5b). In Figure 5c, the study indicated that there was a significant increase in caspase‐9 activity in SNCA‐overexpressed MRC‐5 (19%, *p* < 0.05 vs. empty vector–transfected cells) and A549 (22%, *p* < 0.05 vs. empty vector–transfected cells) cells. For caspase‐12 activity, a significant increase was observed in SNCA‐overexpressed A549 (34%, *p* < 0.05 vs. empty vector–transfected cells) and NCI‐H2170 (44%, *p* < 0.05 vs. empty vector–transfected cells) cells (Figure 5d). For effector caspases, the results showed that after transfection with SNCA for 48 h, caspase‐3/7 activity increased significantly by 36% (*p* < 0.05 vs. empty vector–transfected cells) in A549 cells, whereas there was no change in caspase‐6 activity for the three cell lines (Figure 5e,f). Collectively, these data suggested that SNCA overexpression induced caspase‐8 and caspase‐9 activations in SNCA‐overexpressed MRC‐5 cells, caspase‐3/7, caspase‐8, caspase‐9, and caspase‐12 activations in A549 cells, and caspase‐12 activation in NCI‐H2170 cells.

## 4. Discussion

In this study, we explored the biological relevance of SNCA overexpression in NSCLC cells. Results indicated that SNCA overexpression suppressed cell growth and induced intracellular ROS production in both A549 and NCI‐H2170 cells. Our study indicated that A549 cells are more resistant to SNCA‐induced ROS damage, resulting in less cell death when compared to NCI‐H2170 cells. In PD, it is known that the aggregation of SNCA oligomers ectopically induces cell death and toxicity [33–35]. Few studies revealed that the degradation or depletion of SNCA oligomers could rescue neuronal cell death [36, 37]. However, the roles of SNCA in cancer cells were not clearly understood. ROS plays a significant role in the suppression of various tumors. Anticancer agents including cisplatin, doxorubicin, and Taxol, which modulate ROS generation in cells, have been widely used as cancer therapeutics in multiple cancer types [38–41]. Accumulation of excessive intracellular ROS can trigger oxidative stress, causing DNA, protein, and lipid damage, which can ultimately result in apoptosis [42–44]. Along with our findings, we postulated that the buildup of intracellular oxidative stress induced apoptosis in SNCA‐overexpressed NSCLC cells. Although our findings showed a positive association between ROS production and apoptosis following SNCA overexpression, the causal relationship remains ambiguous, as a functional experiment was not studied. Future studies will include antioxidant rescue experiments using glutathione or N‐acetylcysteine, together with gene modulation assays targeting ROS‐modulating pathways to establish the causal role of ROS in SNCA‐mediated apoptosis [29, 45]. Nevertheless, few studies demonstrated that SNCA regulates multiple apoptotic‐signaling pathways, including the EGFR signaling pathway and PI3K/AKT/mTOR pathway that may interplay with oxidative stress mechanisms [23, 24].

Interestingly, our study has also shown a significant reduction in cell viability and an increase in ROS levels in MRC‐5 cells. This suggests that excessive SNCA overexpression may exert cytotoxic effects in normal lung fibroblasts, potentially contributing to muscle dysfunction. In general, fibroblasts are the predominant stromal cells in muscle tissue [46]. Some studies revealed that the transition of fibroblasts to cancer‐associated fibroblasts contributes to cancer progression via oxidative stress induction [47, 48]. A study by Hart et al. reported that SNCA aggregation is associated with high sarcopenia rates among PD patients due to oxidative stress, neuroinflammation, protein clearance impairment, or mitochondrial dysfunction [49]. Taken together, these findings highlighted the importance of evaluating the roles of SNCA on fibroblast viability and muscle mass maintenance in cancer studies.

Furthermore, our findings indicate that SNCA overexpression induced apoptosis in NSCLC cells. An increased sub‐G1 cell population was observed in SNCA‐overexpressed NSCLC cells through cell cycle analysis. The degraded DNA was identified in the sub‐G1 phase of the cell population [50]. DNA fragmentation is the hallmark of apoptosis [51, 52]. In addition, apoptosis induced by SNCA was evaluated by the caspases′ activities. Based on our study, we revealed that SNCA overexpression activated multiple caspases such as the initiator caspase‐8, caspase‐9, and caspase‐12 in A549 cells and the effector caspase‐3/7. Caspases are the primary drivers of apoptotic cell death regulated by a variety of cellular factors [53]. There are three different ways to initiate cell apoptosis: the extrinsic death receptor pathway, the intrinsic ER stress pathway, and the intrinsic mitochondrion‐dependent pathway [54]. These three pathways lead to the activation of cysteine proteases, that is, caspases. The extrinsic pathway is mediated by the formation of the death‐inducing signaling complex (DISC) followed by caspase‐8 activation [31, 55, 56]. The DISC is a crucial component of the extrinsic apoptosis pathway, where death ligands attach to death receptors on the cell surface [55–57]. In the mitochondria‐dependent apoptotic pathway, Bcl‐2 family proteins play a vital role in regulating mitochondrial outer membrane permeabilization (MOMP) [58]. Subsequently, loss of MOMP results in the release of Cytochrome c, followed by the formation of an apoptosome, which ultimately leads to caspase‐9 activation [55–57]. In addition, caspase‐12 is usually categorized as an inflammatory caspase, and studies have suggested that caspase‐12 participates in ER stress‐induced apoptosis [59–61]. ER stress initiates oxidative stress and translocates caspase‐12 to the cytosol, followed by the activation of caspase‐9 [62, 63]. Consequently, the activation of these initiator caspases shares a common execution step that is regulated by the activation of caspase‐3/7, resulting in apoptosis [56, 57, 62]. This multiple apoptotic activation reflects the complexity of the cellular response to SNCA in A549 cells. A study by Wu et al. revealed that caspase‐8 and caspase‐9 functioned differently during early and late stages of apoptosis induced by mechanical stress [64]. Furthermore, when SNCA was overexpressed in NCI‐H2170 cells, it led to ER stress‐induced apoptosis that was dependent on caspase‐12 but independent on caspase‐3/7. Therefore, gene expression profiling and functional inhibition studies are necessary to harness the proapoptotic potential of SNCA in NSCLC.

Collectively, our study demonstrated that SNCA overexpression induced cell growth inhibition and apoptosis in LUAD A549 cells and LUSC NCI‐H2170 cells, with these effects being dependent on the generation of ROS. By comparing A549 (LUAD) and NCI‐H2170 (LUSC) cells, our results showed that NCI‐H2170 cells are more sensitive to the detrimental effects of SNCA overexpression. Our study has demonstrated elevated cell death, increased production of ROS, greater cell population in the sub‐G1 phase, and late apoptotic stage in SNCA‐overexpressed NCI‐H2170 cells as compared to A549 cells. Moreover, A549 and NCI‐H2170 overexpressing SNCA exhibited distinct caspase activations in apoptosis initiation. The differential responses between LUAD and LUSC can be attributed to their molecular and genomic distinctions [65]. LUAD is generally differentiated from LUSC by the mutations in tyrosine kinase receptors such as *ALK*, *EGFR*, *ROS1*, and *KRAS* [65]. Conversely, CDKN2A, PTEN, NOTCH1, PIK3CA, and FGFR1 mutation frequencies are higher in LUSC [65, 66]. The different biological characteristics between LUAD and LUSC facilitate distinct tumor progression pathways [67–70]. The transcriptomic DNA microarray study by Relli et al. identified 69 distinct tumor prognostic determinants, including key drivers of tumor growth, cell cycle, and metabolic determinants involved in either LUAD or LUSC [69]. Besides, p53 mutations are more common in LUSC, and these mutations lead to the upregulation of oncogenic proteins which results in increased cell growth and proliferation [67, 70].

The molecular and genomic distinctions between LUAD and LUSC may underlie the differential SNCA‐mediated apoptosis and ROS sensitivity observed in our study. LUSC exhibits unique biological behaviors and responses to cellular stress, which could potentially contribute to its distinct vulnerability to SNCA overexpression and the variation in pathway activation compared with LUAD. Taken together, SNCA overexpression has demonstrated potential as a tumor suppressor gene in NSCLC. Future work can focus on studying the molecular changes in SNCA‐overexpressed LUAD and LUSC cells when compared to untreated LUAD and LUSC cells and understanding their physiological differences from the untreated counterparts. Also, in vivo assays can be included to further evaluate the tumor‐suppressive role of SNCA and validate the therapeutic strategies of SNCA‐mediated apoptotic pathways in NSCLC.

In conclusion, SNCA‐overexpressed NSCLC cell model was successfully developed for evaluating the anticancer effects of SNCA. Our results suggested that SNCA overexpression can suppress cell growth by inducing oxidative stress, thereby resulting in apoptosis in NSCLC cells. SNCA overexpression induced caspase‐3/7‐, caspase‐8‐, caspase‐9‐, and caspase‐12‐dependent apoptotic pathways in A549 but only activated caspase‐12‐mediated apoptosis in NCI‐H2170. Also, we revealed the different susceptibilities of LUAD and LUSC cell lines to SNCA overexpression, by which NCI‐H2170 cells are more susceptible to the detrimental effects of SNCA overexpression, resulting in higher ROS generation and notable cell death.

**Nomenclature**
CA: carbonate apatite
CaCl_2_•2H_2_O: calcium chloride dihydrate
DCFH‐DA: 2 ^′^,7 ^′^‐dichlorofluorescein diacetate
DISC: death‐inducing signaling complex
DJ‐1: parkinsonism‐associated deglycase
DMEM: Dulbecco′s Modified Eagle Medium
DMSO: dimethyl sulfoxide
EDTA: ethylenediaminetetraacetic acid
eGFP: enhanced green fluorescent protein
FBS: fetal bovine serum
FBXO7: F‐box Protein 7
HRP: horseradish peroxidase
LRRK2: leucine‐rich repeat Kinase 2
LUAD: lung adenocarcinoma
LUSC: lung squamous cell carcinoma
MOMP: mitochondrial outer membrane permeabilization
MTT: 3‐(4,5‐dimethylthiazol‐2‐yl)‐2,5‐diphenyltetrazolium bromide
NSCLC: non–small cell lung cancer
PBS: phosphate‐buffered saline
PD: Parkinson′s disease
PINK1: PTEN‐induced Kinase 1
SDS‐PAGE: SDS–polyacrylamide gel electrophoresis
SEM: standard error of mean
SNCA: alpha‐synuclein
UCHL1: ubiquitin C‐terminal hydrolase L1

## Author Contributions

**Yong Qi Leong:** data curation, investigation, methodology, project administration, validation, visualization, writing – original draft preparation, writing – review and editing. **Rhun Yian Koh:** supervision, methodology, resources, formal analysis, validation, writing – review and editing. **Christina Gertrude Yap:** supervision, writing – review and editing. **Soi Moi Chye:** resources, writing – review and editing. **Khuen Yen Ng:** conceptualization, funding acquisition, resources, project administration, methodology, supervision, validation, writing – review and editing.

## Funding

This study was funded by the Ministry of Higher Education, Malaysia (10.13039/501100003093, FRGS/1/2019/SKK08/MUSM/02/2) and Monash University Malaysia (10.13039/501100010699, STG‐000045 and STG‐000163). Open access publishing facilitated by Monash University, as part of the Wiley ‐ Monash University agreement via the Council of Australasian University Librarians.

## Conflicts of Interest

The authors declare no conflicts of interest.

## Supporting Information

Additional supporting information can be found online in the Supporting Information section.

## Supporting information


**Supporting Information 1** Figure S1: Green fluorescent signals were observed in empty vector and SNCA‐overexpressed cells by fluorescence microscope. Magnification: 100×; scale bar: 100 *μ*m. SNCA: alpha‐synuclein.

**Supporting Information 2** Figure S2: Representative flow cytometry graphs of cell cycle analysis after 48 h of transfection with CA nanoparticles only, CA/empty vector, or CA/SNCA complexes in (A) MRC‐5, (B) A549, and (C) NCI‐H2170 cells. CA: carbonate apatite; SNCA: alpha‐synuclein.

**Supporting Information 3** Figure S3: Representative flow cytometry charts using Annexin V–FITC/PI staining after 48 h of transfection with CA nanoparticles only, CA/empty vector, or CA/SNCA complexes in (A) MRC‐5, (B) A549, and (C) NCI‐H2170 cells. The charts illustrated the percentage of healthy cells (low left quadrant), early apoptotic cells (lower right quadrant), late apoptotic cells (upper right quadrant), and necrotic cells (upper left quadrant). CA: carbonate apatite; SNCA: alpha‐synuclein.

## Data Availability

The data that support the findings of this study are available on request from the corresponding author. The data are not publicly available due to privacy or ethical restrictions.
