# Supplementary figures and images for: SNCA Overexpression Induces Apoptosis in Non–Small Cell Lung Cancer via Caspase‐Dependent Signaling Pathways
Source: Biomed Res Int. 2026 Apr 15;2026:2222343. doi: 10.1155/bmri/2222343 (PMC13080695; doi:10.1155/bmri/2222343)

(A)

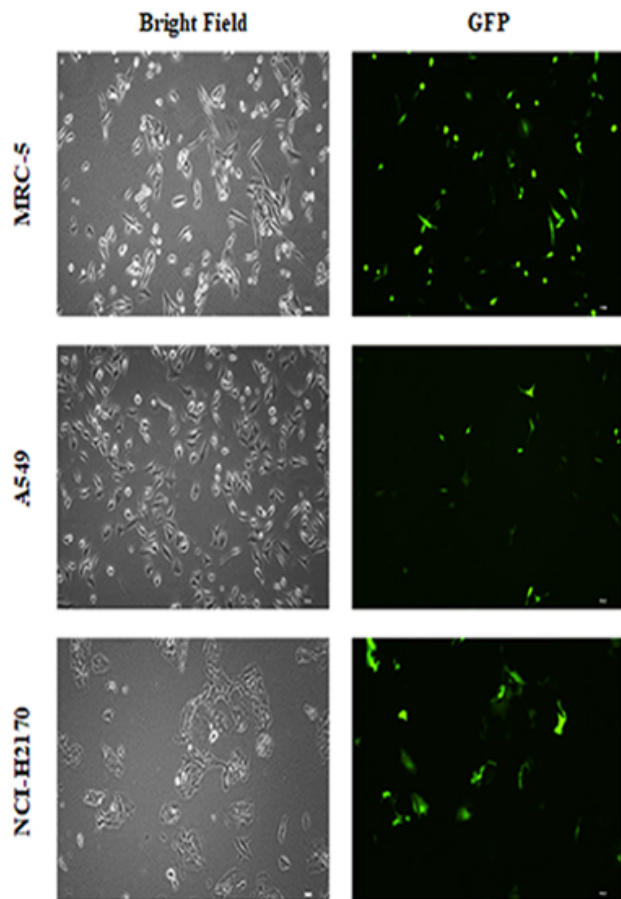

(B)

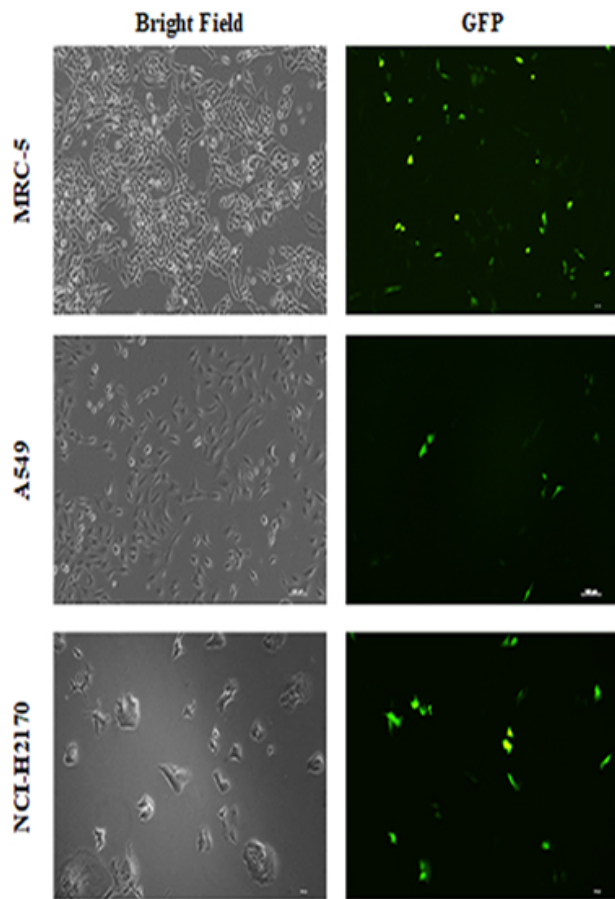

Supplement: Supplementary file 1 — Supporting Information 1 Figure S1: Green fluorescent signals were observed in empty vector and SNCA‐overexpressed cells by fluorescence microscope. Magnification: 100×; scale bar: 100 μm. SNCA: alpha‐synuclein. [file BMRI-2026-2222343-s003.pdf]
