# Supplementary material for: SNCA Overexpression Induces Apoptosis in Non–Small Cell Lung Cancer via Caspase‐Dependent Signaling Pathways
Source: Biomed Res Int. 2026 Apr 15;2026:2222343. doi: 10.1155/bmri/2222343 (PMC13080695; doi:10.1155/bmri/2222343)
Supplement: Supplementary file 2 — Supporting Information 2 Figure S2: Representative flow cytometry graphs of cell cycle analysis after 48 h of transfection with CA nanoparticles only, CA/empty vector, or CA/SNCA complexes in (A) MRC‐5, (B) A549, and (C) NCI‐H2170 cells. CA: carbonate apatite; SNCA: alpha‐synuclein. [file BMRI-2026-2222343-s002.pdf]

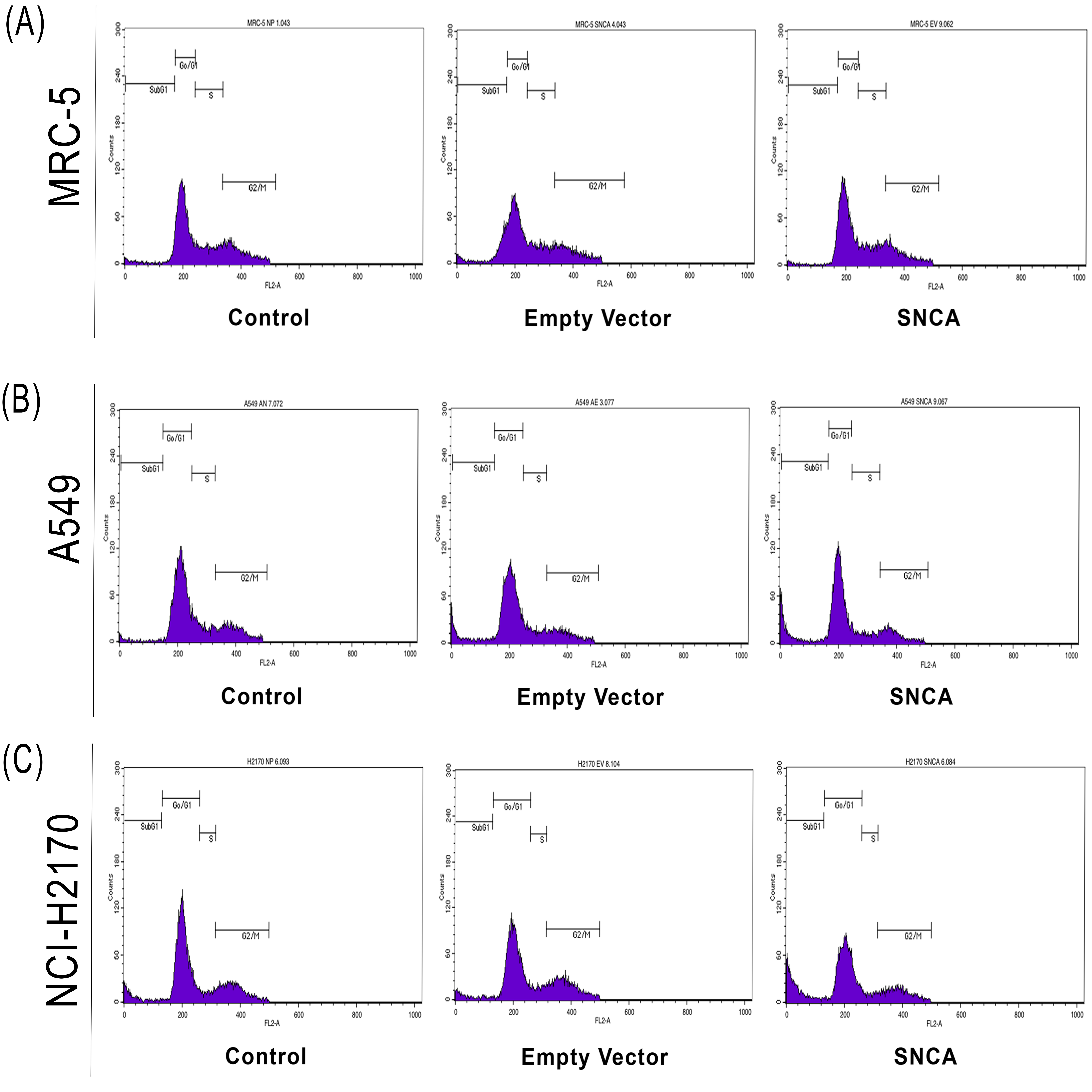
(A)
MRC-5
MRC-5 NP 1.043
MRC-5 SNCA 4.043
MRC-5 EV 9.062
Control
Empty Vector
SNCA
(B)
A549
A549 AN 7.072
A549 AE 3.077
A549 SNCA 9.067
Control
Empty Vector
SNCA
(C)
NCI-H2170
H2170 NP 6.093
H2170 EV 8.104
H2170 SNCA 6.084
Control
Empty Vector
SNCA
SubG1
Go/G1
S
G2/M
Counts
FL2-A
