# Supplementary material for: SNCA Overexpression Induces Apoptosis in Non–Small Cell Lung Cancer via Caspase‐Dependent Signaling Pathways
Source: Biomed Res Int. 2026 Apr 15;2026:2222343. doi: 10.1155/bmri/2222343 (PMC13080695; doi:10.1155/bmri/2222343)
Supplement: Supplementary file 3 — Supporting Information 3 Figure S3: Representative flow cytometry charts using Annexin V–FITC/PI staining after 48 h of transfection with CA nanoparticles only, CA/empty vector, or CA/SNCA complexes in (A) MRC‐5, (B) A549, and (C) NCI‐H2170 cells. The charts illustrated the percentage of healthy cells (low left quadrant), early apoptotic cells (lower right quadrant), late apoptotic cells (upper right quadrant), and necrotic cells (upper left quadrant). CA: carbonate apatite; SNCA: alpha‐synuclein. [file BMRI-2026-2222343-s001.pdf]

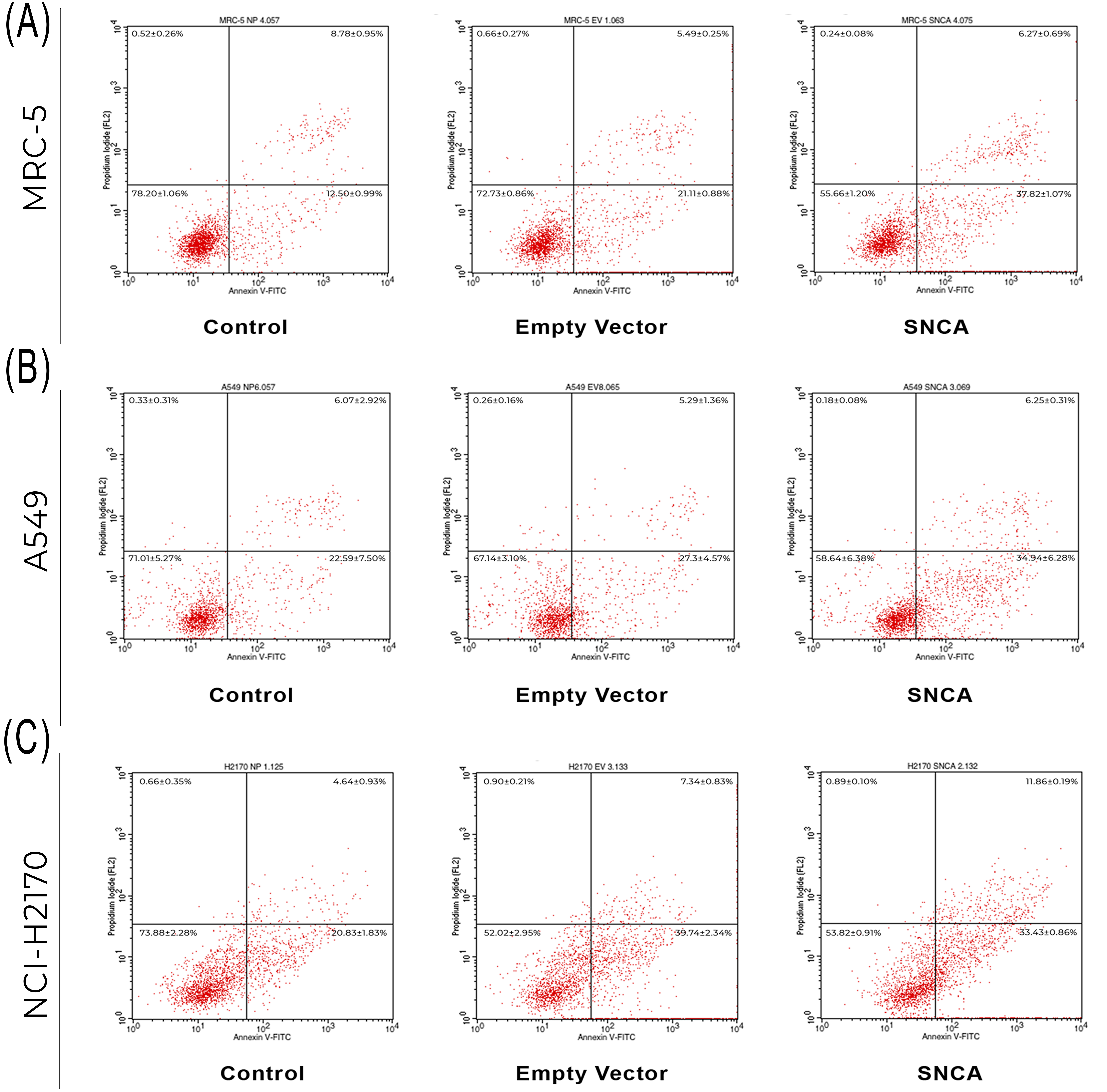

(A)
MRC-5
MRC-5 NP 4.057
0.52±0.26%
8.78±0.95%
78.20±1.06%
12.50±0.99%
MRC-5 EV 1.063
0.66±0.27%
5.49±0.25%
72.73±0.86%
21.11±0.88%
MRC-5 SNCA 4.075
0.24±0.08%
6.27±0.69%
55.66±1.20%
37.82±1.07%
Propidium Iodide (FL2)
Annexin V-FITC
Control
Empty Vector
SNCA
(B)
A549
A549 NP6.057
0.33±0.31%
6.07±2.92%
71.01±5.27%
22.59±7.50%
A549 EV8.065
0.26±0.16%
5.29±1.36%
67.14±3.10%
27.3±4.57%
A549 SNCA 3.069
0.18±0.08%
6.25±0.31%
58.64±6.38%
34.94±6.28%
Control
Empty Vector
SNCA
(C)
NCI-H2170
H2170 NP 1.125
0.66±0.35%
4.64±0.93%
73.88±2.28%
20.83±1.83%
H2170 EV 3.133
0.90±0.21%
7.34±0.83%
52.02±2.95%
39.74±2.34%
H2170 SNCA 2.132
0.89±0.10%
11.86±0.19%
53.82±0.91%
33.43±0.86%
Control
Empty Vector
SNCA
